# Spatiotemporal clustering of cases of Kawasaki disease and associated coronary artery aneurysms in Canada

**DOI:** 10.1038/s41598-018-35848-9

**Published:** 2018-12-05

**Authors:** Jason Hearn, Brian W. McCrindle, Brigitte Mueller, Sunita O’Shea, Bailey Bernknopf, Michael Labelle, Cedric Manlhiot

**Affiliations:** 1 Ted Rogers Centre for Heart Research, Peter Munk Cardiac Centre, University Health Network, University of Toronto, Ontario, Canada; 2 Labatt Family Heart Centre, The Hospital for Sick Children, Department of Pediatrics, Faculty of Medicine, University of Toronto, Ontario, Canada; 3 Cardiovascular Data Management Centre, The Hospital for Sick Children, Department of Surgery, Faculty of Medicine, University of Toronto, Ontario, Canada

## Abstract

Detailed epidemiologic examination of the distribution of Kawasaki disease (KD) cases could help elucidate the etiology and pathogenesis of this puzzling condition. Location of residence at KD admission was obtained for patients diagnosed in Canada (excluding Quebec) between March 2004 and March 2015. We identified 4,839 patients, 164 of whom (3.4%) developed a coronary artery aneurysm (CAA). A spatiotemporal clustering analysis was performed to determine whether non-random clusters emerged in the distributions of KD and CAA cases. A high-incidence KD cluster occurred in Toronto, ON, between October 2004 and May 2005 (116 cases; relative risk (RR) = 3.43; p < 0.001). A cluster of increased CAA frequency emerged in Mississauga, ON, between April 2004 and September 2005 (17% of KD cases; RR = 4.86). High-incidence clusters also arose in British Columbia (November 2010 to March 2011) and Alberta (January 2010 to November 2012) for KD and CAA, respectively. In an exploratory comparison between the primary KD cluster and reference groups of varying spatial and temporal origin, the main cluster demonstrated higher frequencies of conjunctivitis, oral mucosa changes and treatment with antibiotics, suggesting a possible coincident infectious process. Further spatiotemporal evaluation of KD cases might help understand the probable multifactorial etiology.

## Introduction

Kawasaki disease (KD) is an acute, mucocutaneous illness of unknown etiology^1^. First reported in 1967^2^, KD primarily affects children younger than 5 years of age and is indicated by a constellation of clinical signs including vasculitis, which can lead to arterial complications^3^, namely coronary artery aneurysms (CAA)^4^. Although the likelihood of CAA has been significantly reduced with the use of prompt treatment with intravenous immunoglobulin^5^, this complication remains the primary cause of morbidity and mortality associated with KD^6^. Given uncertainty regarding the etiology and specific treatment, efforts are ongoing to elucidate the genetic, environmental and infectious causes of KD and the optimal strategies to prevent complications^7–9^.

While the genetic susceptibility for KD within certain population groups is well-documented, with the observation of consistently-higher incidence rates amongst those of East Asian descent^10^, the possible environmental and infectious causes remain unclear. The documented seasonality of KD^11^ and its frequent co-occurrence with infection^12,13^ has led many to conclude that KD is associated with an environmental and/or infectious trigger inciting an extreme inflammatory response in genetically-susceptible children^14^. Unfortunately, the trigger (or triggers) remain(s) poorly understood, as researchers continue to debate whether the causal agent is infectious, environmental or some combination of the two. Furthermore, though previous research has shown the development of CAA in KD patients to be unlinked to the presence of an infection^15^, the environmental causes of poor coronary artery outcomes have yet to be thoroughly investigated. Thus, we explored the spatiotemporal distribution of both KD cases and CAA complications in Canada between March 2004 and March 2015, with the goal of identifying non-random clusters that might help explain the association between occurrence and infectious and/or environmental phenomena.

## Results

### Descriptive Analysis

**Figure 1:**
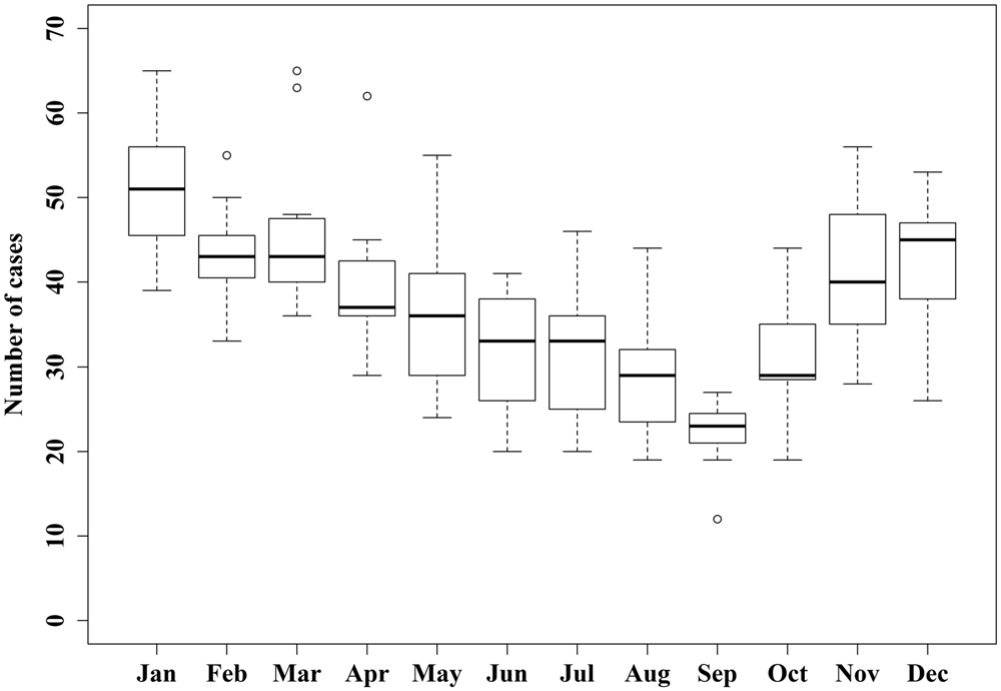
Annual distribution of Kawasaki disease cases in Canada between March 2004 and March 2015.

A total of 4,839 KD patients (2,857 male/1,982 female) were eligible for inclusion in the study, 164 of whom (3.4%, 115 male/49 female) developed CAA. The annual incidence of KD for the study period was 7.7 cases per 100,000 children aged 19 years or younger. The vast majority of KD and CAA cases occurred in children less than 5 years of age (incidence = 20.5 cases per 100,000 children aged 5 years or younger). Based on the sex distributions of both KD and CAA, the frequency of CAA in KD patients was found to be significantly higher amongst males (4.0% vs. 2.5%, p = 0.005). Geographically, approximately 60% of both KD and CAA cases were treated in Ontario, whereas Alberta demonstrated the highest frequency of CAA in KD patients at 6.0%. Figure 1 demonstrates the apparent seasonal pattern of KD cases during the study period.

### Spatiotemporal Clustering Analysis

**Figure 2:**
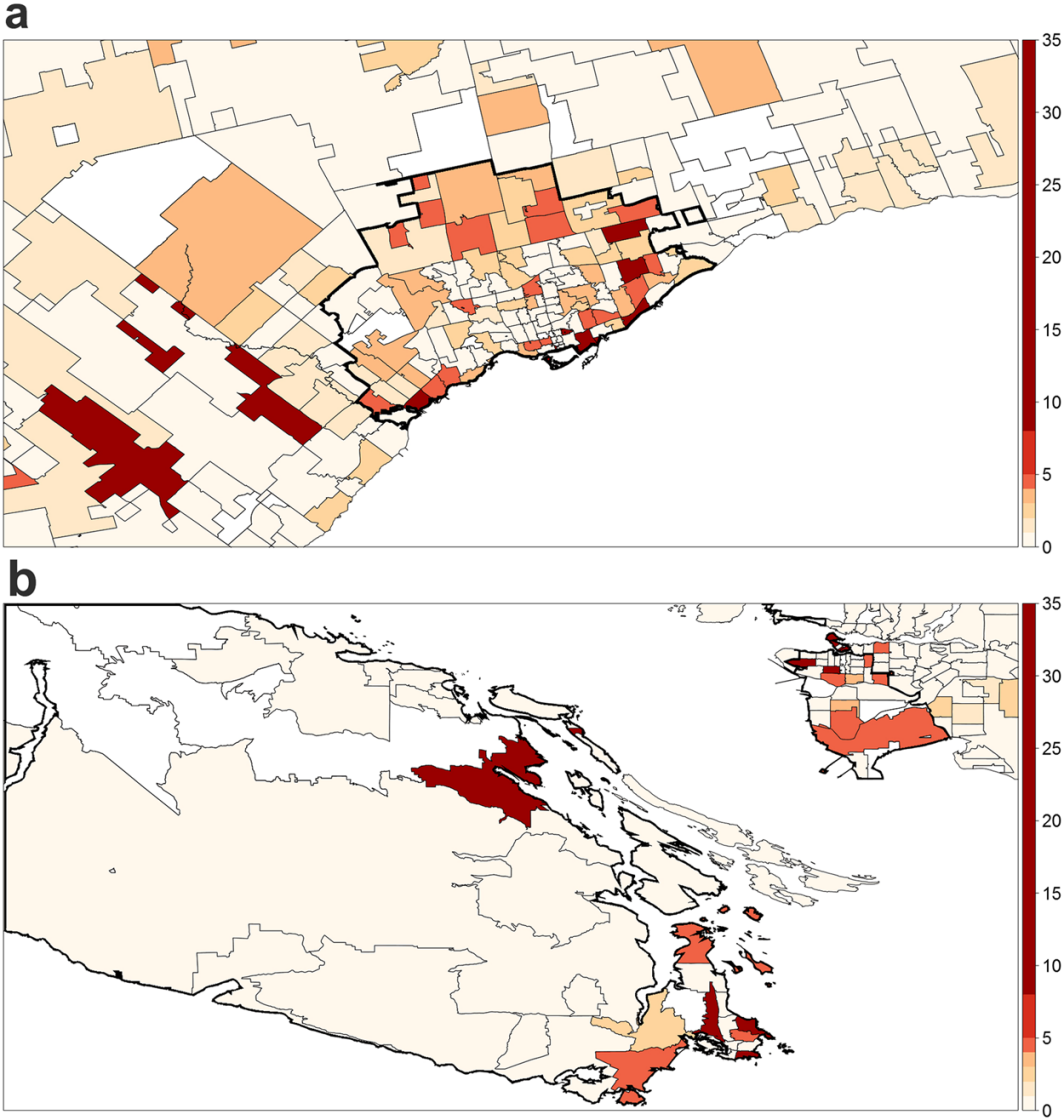
Major spatiotemporal clusters of high KD incidence identified in Canada between March 2004 and March 2015, where the colour scale represents relative risk amongst individuals below 19 years of age. (**a**) Toronto, ON – Oct. 2004 to May 2005 (**b**) Port Renfrew, BC – Nov. 2010 to Mar. 2011 (note: part of cluster not shown due to sparsely-population areas).

The spatiotemporal clustering analysis revealed several clusters of increased or decreased event incidence. The major KD cluster of high incidence occurred in and around Toronto, ON, between October 2004 and May 2005, with 116 patients being diagnosed with KD (incidence = 26.1 per 100,000 children aged 19 years or younger; relative risk (RR) = 3.43; p ≤ 0.001; see Fig. 2a). During this seven-month time frame, 32% of all KD cases took place in the spatial window surrounding Toronto, an area that accounts for only 9.9% of the child population in Canada. Furthermore, 25 cases of KD were reported within a 120-kilometer radius centered in Port Renfrew, BC, during a four-month period starting in November 2010 (32.9 per 100,000 children aged 19 years or younger; RR = 4.27; p = 0.01; see Fig. 2b). The spatial window accounted for 11% of all KD cases during the clustering time, despite only 3.3% of Canadian children living in the area. For a complete summary of the identified KD clusters, see Table 1.

**Table 1:** Clusters of high KD incidence identified in Canada between 2004 and 2015.

Centre Point	Radius (km)	Start Date	End Date	Population	Obs.	Exp.	Incidence	RR
Toronto, ON	23.0	Oct. 2004	May 2005	767,000	116	34.4	26.1	3.43
All Canada		Oct. 2010	Apr. 2011	5,679,165	337	219.0	11.9	1.58
Port Renfrew, BC	119.5	Nov. 2010	Mar. 2011	231,105	25	5.88	32.9	4.27
Calgary, AB	23.6	Oct. 2007	Apr. 2008	116,980	18	4.5	30.7	3.98

Obs.: number of observed KD cases; Exp.: number of expected KD cases;

Incidence: KD cases per 100,000 children aged 19 years or younger; RR: relative risk.

**Figure 3:**
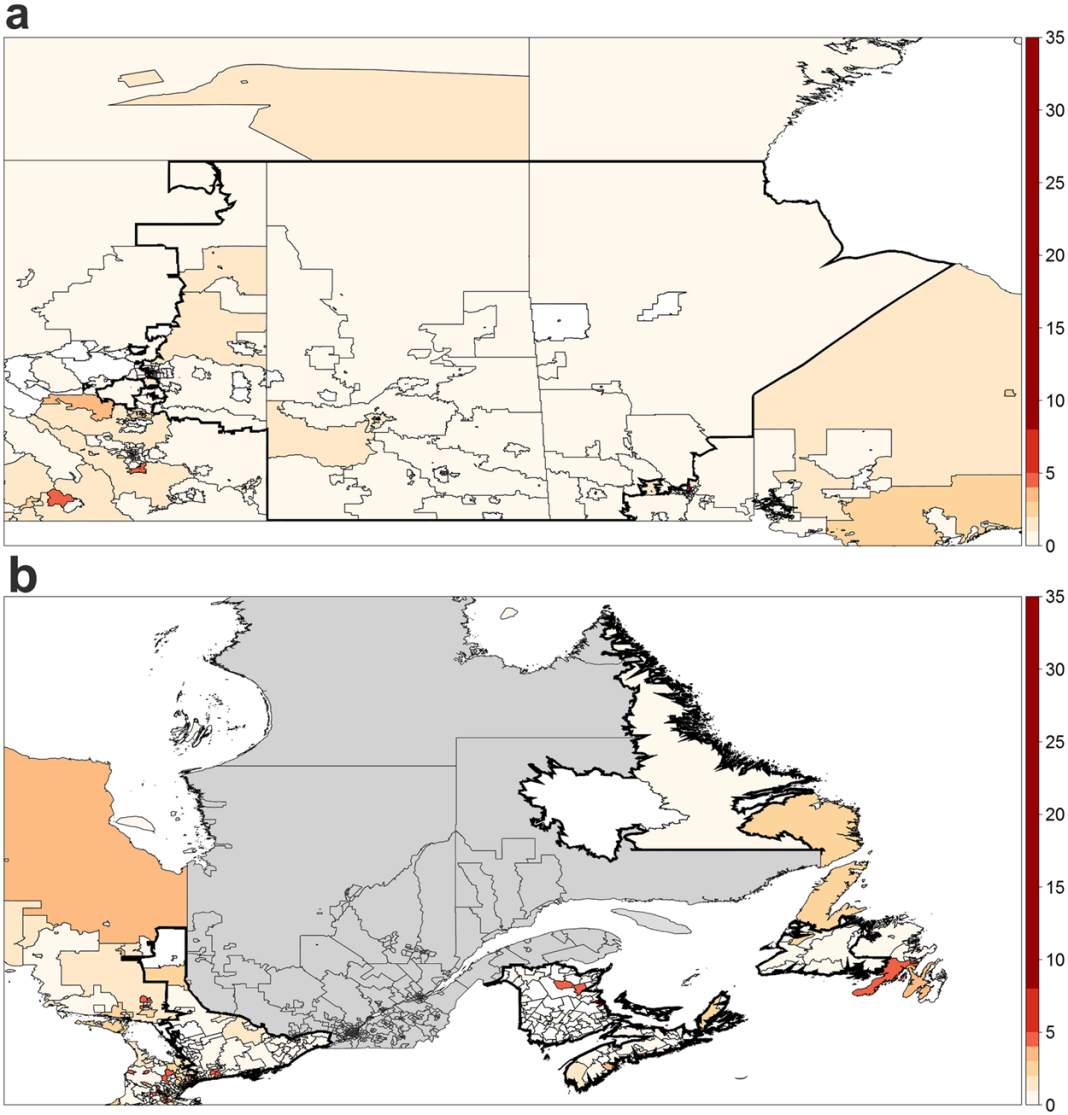
Major spatiotemporal clusters of low KD incidence identified in Canada between March 2004 and March 2015, where the colour scale represents relative risk amongst individuals below 19 years of age. (**a**) Prince Albert, SK – Aug. 2006 to Jun. 2008 (**b**) Edmundston, NB – Jun. 2007 to Nov. 2008 (note: grey indicates lack of available data in Quebec).

Though some areas demonstrated an increased KD incidence during a particular time window, others showed clusters of significantly-reduced incidence of KD (as seen in Table 2). The major cluster of low KD incidence occurred in the area around Prince Albert, SK, between August 2006 and June 2008, where only 20 patients were diagnosed with KD (incidence = 1.7 per 100,000 children aged 19 years or younger; RR = 0.22; p ≤ 0.001; see Fig. 3a). Despite containing 8.8% of the Canadian child population, only 2.9% of KD cases during the temporal window occurred in the area around Prince Albert. Between June 2007 and November 2008, only 53 KD cases (incidence = 3.5 per 100,000 children aged 19 years or younger; RR = 0.45; p = 0.002; see Fig. 3b) occurred within a 943-kilometer radius centered in Edmundston, NB. Although the children in this area constitute about 15.3% of the Canadian child population, only 10.7% of cases in the 17-month period occurred in the spatial window.

**Table 2:** Clusters of low KD incidence identified in Canada between 2004 and 2015.

Centre Point	Radius (km)	Start Date	End Date	Population	Obs.	Exp.	Incidence	RR
Prince Albert, SK	643.6	Aug. 2006	Jun. 2008	630,825	20	89.5	1.7	0.22
All Canada		Jun. 2007	Nov. 2007	5,679,165	99	184.1	4.2	0.53
Edmundston, NB	942.6	Jun. 2007	Nov. 2008	1,053,035	53	115.8	3.5	0.45
Amherstburg, ON	326.6	Apr. 2014	Sept. 2014	1,180,385	10	38.3	2.0	0.26

Obs.: number of observed KD cases; Exp.: number of expected KD cases;

Incidence: KD cases per 100,000 children aged 19 years or younger; RR: relative risk.

In addition to the spatiotemporal clusters of KD, two purely temporal clusters (i.e. a national spatial window) emerged in the clustering analysis. A period of high KD incidence occurred between October 2010 and April 2011, when approximately 337 cases of KD (incidence = 11.9 per 100,000 children aged 19 years or younger, RR = 1.58, p ≤ 0.001; see Table 1) were recorded throughout the country. The analysis also identified a low-incidence cluster between June 2007 and November 2007, when only 99 Canadian children were diagnosed with KD (incidence = 4.2 per 100,000 children aged 19 years or younger, RR = 0.53, p ≤ 0.001; see Table 2).

**Figure 4:**
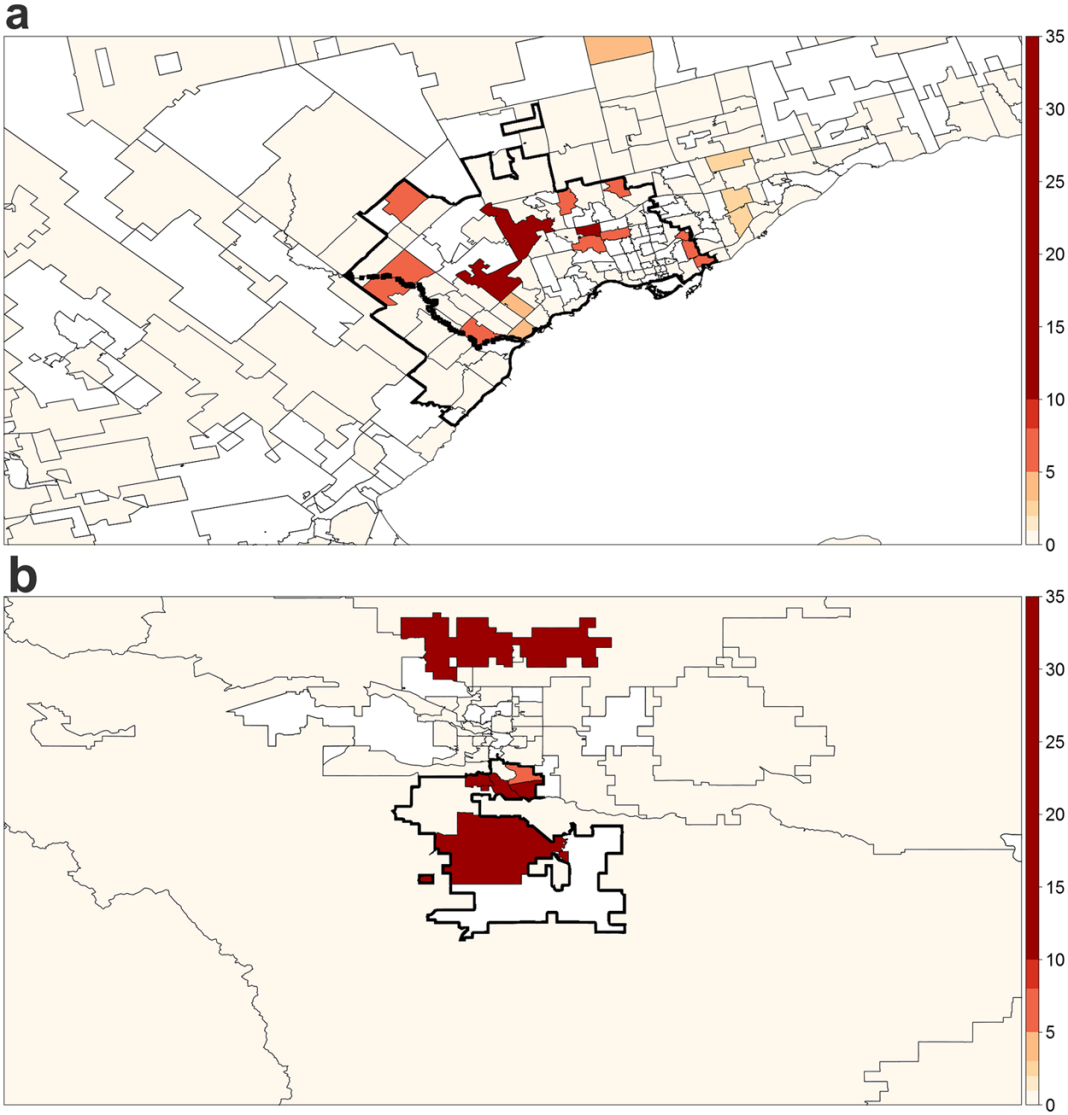
Major spatiotemporal clusters of increased CAA frequency in KD patients identified in Canada between March 2004 and March 2015, where the colour scale represents relative risk amongst individuals diagnosed with KD. (**a**) Mississauga, ON – Apr. 2004 to Sept. 2005 (**b**) Okotoks, AB – Jan. 2010 to Nov. 2012.

The spatiotemporal analysis also revealed two major clusters of increased CAA frequency in the study window. Firstly, CAA developed in 17 of 102 (17%) KD cases in the area surrounding Mississauga, ON, between April 2004 and September 2005 (RR = 4.86; Fig. 4a). Moreover, 7 of 18 (39%) KD patients developed CAA in the 26-kilometer radius around Okotoks, AB, between January 2010 and November 2012 (RR = 10.9; Fig. 4a). Due to the relatively-small number of CAA cases during the study period, statistical significance of these clusters could not be reliably assessed. For a complete summary of the identified CAA clusters, see Table 3.

**Table 3:** Clusters of increased CAA frequency in KD patients identified in Canada between March 2004 and March 2015.

Centre Point	Radius (km)	Start Date	End Date	KD Cases	Obs	Exp	RR
Mississauga, ON	21.9	Apr. 2004	Sept. 2005	102	17	3.8	4.86
Okotoks, AB	25.6	Jan. 2010	Nov. 2012	18	7	0.7	10.9
Nunavut	1915.9	Nov. 2006	May 2009	9	5	0.3	15.5
Markham, ON	6.3	Apr. 2011	Jan. 2012	8	5	0.3	15.2

Obs.: number of observed CAA cases; Exp.: number of expected CAA cases;

RR: relative risk.

Lastly, as most KD cases occur in young children, a sensitivity analysis was performed to determine whether restricting the study population to patients younger than 5 years of age would change our identified clusters. In repeating the spatiotemporal analyses for the narrowed patient population, the emerging clusters remained unchanged for both high incidence of KD and high frequency of CAA.

### Epidemiologic Comparison

Epidemiologic and clinical characteristics of the primary KD cluster (Toronto, ON, between October 2004 and May 2005) were compared with four reference groups of varying spatial and temporal origin (RG1, RG2, RG3 and RG4; detailed in the Methods section). As shown in Table 4, several differences were noted in the exploratory comparison of the main cluster with RG1, RG3 and RG4. When compared to these three reference groups, patients in the main cluster demonstrated higher frequencies of conjunctivitis (p = 0.04, p = 0.002, p = 0.08, respectively), oral mucosa changes (p < 0.001, p = 0.16, p < 0.001, respectively) and, to a lesser extent, antibiotic treatment (p = 0.11, p = 0.08, p = 0.11, respectively) – all of which may be suggestive of an infectious process. The only reference group that did not follow these patterns was RG2 (i.e. the same spatial area, but in the year prior to the main cluster), which showed negligible differences in most of the investigated variables when compared to the main cluster.

**Table 4:** Epidemiologic comparison of the major KD cluster (i.e., Toronto, ON, from October 2004 to May 2005) and four populations outside of the cluster in Ontario.

	Main Cluster	Same Time, Outside Area		Same Area, Year Prior		Same Area, Year After		Random Subset	
		*Value*	*P*	*Value*	*P*	*Value*	*P*	*Value*	*P*
Demographic									
Number of KD cases	104	112		52		69		104	
Sex, male	70/104 (67%)	75/112 (67%)	1.00	35/52 (67%)	1.00	43/69 (62%)	0.52	71/104 (68%)	1.00
Age at diagnosis (years)	4.1 ± 2.7	3.3 ± 2.6	0.026	3.1 ± 2.9	0.048	3.7 ± 2.6	0.42	3.4 ± 2.9	0.078
Clinical									
Incomplete KD	33/99 (33%)	52/101 (52%)	0.01	9/47 (19%)	0.083	23/61 (38%)	0.61	37/92 (40%)	0.37
Conjunctivitis	90/101 (89%)	86/110 (78%)	0.041	44/48 (92%)	0.77	44/64 (69%)	0.002	80/100 (80%)	0.082
Cervical lymphadenopathy	57/101 (56%)	44/110 (40%)	0.019	28/48 (58%)	0.86	35/64 (55%)	0.87	52/101 (52%)	0.57
Oral mucosa changes	91/101 (90%)	77/110 (70%)	<0.001	43/48 (90%)	1.00	52/64 (81%)	0.16	70/101 (69%)	<0.001
Rash	80/101 (79%)	83/110 (76%)	0.62	47/49 (96%)	0.007	52/64 (81%)	0.84	80/101 (79%)	1.00
Edema/peeling extremities	73/101 (72%)	67/110 (61%)	0.11	35/48 (73%)	1.00	41/64 (64%)	0.3	62/101 (61%)	0.13
Length of initial fever (days)	6 ± 2 (N = 96)	7 ± 3 (N = 99)	0.20	7 ± 3 (N = 44)	0.30	7 ± 4 (N = 59)	0.25	6 ± 3 (N = 92)	0.80
Total length of fever (days)	8 ± 2 (N = 97)	7 ± 3 (N = 91)	0.73	8 ± 3 (N = 43)	0.27	8 ± 4 (N = 63)	0.22	7 ± 3 (N = 89)	0.76
Treatment									
Aspirin	94/100 (94%)	101/110 (92%)	0.60	45/50 (90%)	0.51	62/68 (91%)	0.55	96/101 (95%)	0.77
IVIG	97/99 (98%)	104/110 (94%)	0.29	45/50 (90%)	0.043	63/68 (93%)	0.12	98/103 (95%)	0.45
Multiple IVIG	13/99 (13%)	7/110 (6%)	0.11	2/50 (4%)	0.092	3/68 (4%)	0.067	9/103 (9%)	0.37
Steroids	5/88 (6%)	2/91 (2%)	0.27	5/47 (11%)	0.32	2/57 (4%)	0.70	5/88 (6%)	1.00
Antibiotics	48/98 (49%)	37/99 (37%)	0.11	27/50 (54%)	0.60	23/66 (35%)	0.08	36/98 (37%)	0.11
Coronary artery aneurysm	15/87 (17%)	16/97 (16%)	1.00	16/49 (33%)	0.055	9/60 (15%)	0.82	16/81 (20%)	0.70
Giant + non-giant aneurysm	2/87 (2%)	3/96 (3%)	1.00	1/49 (2%)	1.00	1/60 (2%)	1.00	0/81 (0%)	0.50
Length of hospital stay (days)	5 ± 4 (N = 98)	4 ± 3 (N = 106)	0.38	6 ± 5 (N = 47)	0.34	4 ± 2 (N = 65)	0.12	5 ± 5 (N = 98)	0.96

IVIG: intravenous immunoglobulin, KD: Kawasaki disease, P: a comparison between a given value and the corresponding value in the main cluster

In an attempt to explain the apparent infectious process, we searched reportable disease databases – made available by both Public Health Ontario^16^ and the British Columbia Centre for Disease Control^17^ – for an infectious disease demonstrating increased incidence during the two major KD clusters. After thorough examination of the two databases, no increased prevalence of any single infectious disease was found to coincide with the two clusters of high KD incidence.

## Discussion

The presence of space-time clusters noted in this study adds to the growing body of evidence associating the etiology and pathophysiology of KD with localized, infectious and/or environmental phenomena. First, it is worth noting that all four of the identified KD clusters occurred in and around the winter season. This result, in conjunction with the apparent seasonal pattern of KD presented in Fig. 1, is consistent with previous evidence of a possible KD etiologic agent present in increased concentrations during winter months in the Northern Hemisphere^11^. The geographic location of the two major KD clusters also offers potential insight regarding the condition’s etiology. The presence of a primary cluster in Toronto, ON, for example, supports previously-reported KD risk factors associated with living in an urban setting^14^, and the presence of a localized infectious agent (which is further discussed in the subsequent section). Moreover, this cluster is also consistent with the previously-reported increased genetic susceptibility in people of Asian ancestry, as the city’s Asian population (representing approximately 38% of the population) is significantly larger than that of the rest of the country (only 15%, p ≤ 0.001)^18^. Lastly, the Port Renfrew, BC, cluster supports the reported link between an increased KD incidence and westerly winds over the Pacific Ocean – wind patterns that have been previously associated with elevated concentrations of fungal particles in the atmosphere^19,20^. The potential effect of these wind patterns was further supported by the presence of low-incidence clusters in the Prairies and in the Eastern provinces – areas far removed from westerly wind formations.

Though clusters of increased frequency were also noted in the spatiotemporal analysis of CAA, the underlying causes were less easily extracted from the results given the relatively-low number of CAA cases. As reported previously in the literature, the number of CAA cases was likely an underestimation due to its basis on absolute internal lumen diameter and not a relative index, such as coronary artery z-scores^21^. Unlike the KD incidence analysis, no consistent seasonal pattern appeared to emerge in the spatiotemporal analysis of the frequency of CAA. Given the association between KD seasonality and the increased prevalence of an infectious agent, the apparent lack of CAA seasonality reinforces McCrindle *et al*. and their claim that the development of CAA is not associated with the presence of a concomitant infection^15^. The degree of both spatial and temporal overlap between the major CAA frequency cluster (in Mississauga) and KD incidence cluster (in Toronto) also suggests a potential commonality between the causal factors. To avoid this result being discounted, it is worth noting that – as per the study design – an increased KD incidence was not necessarily associated with an increased CAA frequency, as frequency was defined on a *per KD case* basis.

The results of the exploratory epidemiologic comparison offer important insight regarding the potential source of a localized KD trigger. The main KD cluster demonstrated important differences when compared to three of the four reference groups, including higher frequencies of conjunctivitis, oral mucosa changes and antibiotic treatment. Not all of these differences were found to be statistically significant, however, the differences were consistent and relatively large across the three variables, even with the limited number of cases in each group. Seeing as conjunctivitis and oral mucosa changes are manifestations commonly associated with infection, and antibiotics are commonly used to treat infection, the demonstrated patterns suggest the potential presence of an infectious trigger in the main cluster. Moreover, as RG2 showed negligible differences when compared to the main cluster, it is possible that the infectious trigger was already present in the geographic area during the year prior to the main cluster, but perhaps at a lower concentration. While no single infectious disease was found to coincide with the major KD clusters in Ontario and British Columbia, the examination was limited to diseases included in each province’s reportable disease database. Thus, we cannot discount the potential presence of an infectious trigger that is not routinely reported to public health authorities.

Due to the retrospective nature of the research study, the spatiotemporal analysis and epidemiologic comparison were limited to data available from the Canadian Institute for Health Information, The Hospital for Sick Children and the 2011 Canadian Census – all of which were assumed to be accurate and complete. The spatiotemporal analysis of CAA was limited by the relatively-small number of cases occurring in Canada during the study window. Specifically, the small sample size made it difficult to scan for clusters of low CAA frequency, as this analysis would simply isolate the many regions in which a CAA complication did not occur in a given time window. The accuracy of the spatiotemporal analysis was also limited by the moderate resolution of the available geographic data, which identified a patient’s place of residence as the centre point of their forward sortation area (FSA) at time of admission. Though FSA is often an effective proxy for location, specifically in densely-populated areas, it offers suboptimal resolution for large geographic and sparsely-populated areas. The exploratory epidemiologic analysis was limited to clusters that coincided with a major KD surveillance study performed by The Hospital for Sick Children in Ontario between 1995 and 2006^22^. Fortunately, the main KD cluster fell within the identified window, minimizing the effect of this limitation. Lastly, the results of the epidemiologic comparison should be interpreted with caution, given that the identified patterns emerged in a post hoc analysis. Future work could aim to strengthen the claim of a potential coincident infectious process in regions of high KD incidence by performing a prospective epidemiologic study.

To conclude, the etiology and pathophysiology of KD and its associated complications remain incompletely understood. To determine the potential environmental and infectious causes of this condition, we performed a spatiotemporal analysis of KD cases and associated CAA complications occurring in Canada between 2004 and 2015. High-incidence KD clusters were identified in Toronto, ON, between October 2004 and May 2005, as well as in the area surrounding Port Renfrew, BC, between November 2010 and March 2011. Clusters of low KD incidence were noted in and around Prince Albert, SK, between August 2006 and June 2008, and near Edmundston, NB, between June 2007 and November 2008. Both the spatial and temporal distributions provide support for current hypotheses regarding the multifactorial cause of KD, including heightened risk in winter months, genetic susceptibility in Asian populations, and the increased incidence in areas experiencing westerly wind patterns. Increased frequency of CAA complications in KD patients were identified in Mississauga, ON, between April 2004 and September 2005, and in Okotoks, AB, between January 2010 and November 2012. The apparent lack of seasonality in the distribution of CAA complications supports previous hypotheses that the development of CAA in KD patients is not associated with the presence of an infection. Lastly, an exploratory epidemiologic analysis revealed that, when compared to three reference groups, patients in the main KD cluster showed higher frequencies of conjunctivitis, oral mucosa changes and antibiotic treatment – all of which may be suggestive of an infectious process. While the results of the exploratory comparison suggest the potential presence of an infectious trigger in the main cluster, the exact source of the trigger remains unknown.

## Methods

### Data Sources

This study was approved by the Research Ethics Board (REB) at The Hospital for Sick Children and study procedures were carried out following the approved protocol in accordance with the Tri-Council Policy Statement on Ethical Conduct for Research Involving Humans 2010 (TCPS 2), Ontario and Canadian law. Under TCPS 2, a waiver of consent was granted by the REB for the following reasons: a very large study population and patients too difficult to locate. Descriptive data were extracted from a dataset originally collected by the Canadian Institute for Health Information for all KD patients diagnosed and hospitalized in Canada (not including Quebec) between March 2004 and March 2015. It is worth noting that this dataset is generally considered complete, and that the methods used in its collection have been thoroughly documented in the literature^23^. The primary inclusion criterion was a hospital admission associated with a diagnostic code (ICD-10-CA standard) of M30.3 (mucocutaneous lymph node syndrome [KD]), excluding inter-hospital transfers during the acute phase, acute readmissions, admissions for procedures/complications during follow-up and admissions not related to KD. Multiple hospital admissions for the same patient were deemed to be separate incident cases only if they were at least eight weeks apart and both were associated with intravenous immunoglobulin treatments; all other admissions were considered to be readmissions associated with the original event. CAA status was assigned upon discharge from the hospital following the original KD admission, as CAA generally develops during the acute phase of KD. Given the nature of this disease, there is little reason to think that substantial under-recording of CAA after March 2015 occurred. Complete details on the generation of the clinical dataset used in this study have been previously reported^23^.

The following parameters were obtained for each patient: the date of KD diagnosis; the sex, age and location of residence at diagnosis; and a binary indicator of whether or not the patient developed CAA as a result of their illness. Location of residence was provided in terms of the patient’s FSA (i.e., the first three characters of their postal code) at the time of diagnosis. In addition to the nationwide distribution data, detailed epidemiologic and clinical data for KD cases occurring in Ontario were also obtained from a series of triennial surveillance studies performed by researchers at The Hospital for Sick Children between 1995 and 2006^22^. Lastly, the age and sex demographics of each FSA were obtained from the 2011 Canadian Census^24^, and used to assess the population at risk of developing KD. The 2011 census was chosen in place of the 2006 or 2016 censuses, as it was considered to be most representative of the 2004–2015 study period. Lastly, the FSA boundary file was obtained from the 2011 Canadian Census^25^, and used to spatially locate individual FSAs.

### Study Population

In total, data were collected for 4,924 patients diagnosed and hospitalized with KD. A total of 85 patients were excluded based on their reported place of residence. The excluded patients either had their place of residence listed as a province (and not a specific FSA), an FSA in Quebec (for which we had minimal data), or a location in the United States of America (which fell outside the scope of this study).

### Data Pre-Processing

First, the at-risk population for KD was identified as the number of persons aged 19 years or younger living in a given area. Accordingly, a *KD population file* was generated containing the number of children living in each FSA at the time of the 2011 Canadian Census. The population at risk of developing CAA, on the other hand, was identified as the number of patients diagnosed with KD in a given area at a given point in time. Thus, a second, *CAA population file* was generated containing the number of patients diagnosed with KD in each FSA in a given month. Next, a *case file* was generated containing the diagnosis date and FSA of residence for each KD case, as well as a binary indicator of whether CAA developed. Patients were classified as being affected by coronary artery aneurysms if they had a diagnostic code I25.4 (Coronary artery aneurysm or coronary arteriovenous fistula, acquired; clinical definition: abnormal balloon- or sac-like dilatation in the wall of coronary vessels most often due to coronary atherosclerosis or inflammatory disease such as Kawasaki disease) associated with any of their hospital admissions. Lastly, a *geography file* was generated containing the longitude and latitude coordinates at the center point of each FSA.

### Spatiotemporal Clustering Analysis

Using the population, case and geography files generated in the pre-processing phase, discrete Poisson models were fitted and tested using the SaTScan software and Kuldorff’s spatial scan statistic^26^. This statistic is defined as a cylindrical window with a circular base representing a geographic location and a height corresponding to time. As the window is allowed to vary in terms of its height, diameter and spatial location, the statistic essentially visits each possible time period for each possible geographic location and size. From the vast number of generated cylinders spanning the entire study region (both in time and space), clusters of increased or decreased event incidence are identified and outputted from the SaTScan software^27^.

For the purposes of this study, the maximum spatial cluster size was identified as 25% of the area of interest for both the KD and CAA analyses. KD clusters of both increased and decreased incidence were identified, whereas only clusters of high frequency were analyzed for CAA. The maximum temporal cluster sizes were set as 24 months for the KD analysis and 36 months for the CAA analysis. The CAA analysis included only clusters of increased frequency and a larger temporal cluster size due to the reduced number of CAA cases taking place in the study region. Log likelihood ratio tests were performed to assess the significance of space-time clusters, with p-values being obtained from a 999-iteration Monte Carlo simulation. All clustering analyses were performed in SaTScan v9.4.4, while visual interpretation of the outputted clusters was completed using the R Project for Statistical Computing v3.4.2.

### Epidemiologic Comparison

Once the spatiotemporal analysis had been completed, it was found that one of the major KD clusters occurred in Ontario between October 2004 and May 2005. Given the overlap between this cluster and the aforementioned Ontario KD surveillance data (available for cases between 1995 and 2006)^22^, an exploratory epidemiologic analysis was subsequently performed on the identified cluster. Specifically, cases that occurred within the identified space-time window were designated as the *main cluster*, and were individually compared to the following groups of cases occurring outside of the cluster:

- RG1: Cases occurring within the same temporal window, but outside the spatial window,
- RG2: Cases occurring within the same spatial window, but within the equivalent temporal window a year prior to the identified cluster (i.e. October 2003 to May 2004),
- RG3: Cases occurring within the same spatial window, but within the equivalent temporal window a year after the identified cluster (i.e. October 2005 to May 2006), and
- RG4: A random subset of cases occurring within two years of the temporal window.

To assess the similarities and differences between the main cluster and the four reference groups, various demographic, clinical and treatment variables were examined. The mean and standard deviation were reported for continuous parameters, whereas categorical variables were presented in terms of count and percentage. With the goal of identifying important differences between the main cluster and the four reference groups, the significance of the difference from the main cluster was reported for each reference-group parameter. All analyses were performed using the R Project for Statistical Computing v3.4.2.

## Data Availability

Both the Hospital for Sick Children Ethics Committee and the Canadian Institute for Health Information have placed legal restrictions on sharing the data used in this study. The data from this study contains personal health information and as such, disclosure and distribution, even in an anonymized format, is restricted under the Ontario Personal Health Information Protection Act (PHIPA). Some data will only be available until 2019-MAR-31 after which it must be destroyed as per the Non-Disclosure/Confidentiality Agreement required by the Canadian Institute for Health Information. Data used in this study can be accessed by qualified researchers who meet the criteria for access to confidential data. In addition to contacting the study PI to access the data, requestors will be required to obtain approval from the Hospital for Sick Children Ethics Committee (reb.admin@sickkids.ca) and the Canadian Institute for Health Information Data Access Program (https://www.cihi.ca/en/access-data-and-reports/make-a-data-request).
